# Acceptor-doping of lead-free (Ba_0.82_Ca_0.18_)(Zr_0.08_Ti_0.92_)O_3_ with Fe induces piezoelectric hardening

**DOI:** 10.1039/d5ma01411e

**Published:** 2026-02-19

**Authors:** Anna M. Paulik, Anamaria Mihaljević, Kriti Batra, Arpad M. Rostas, Emre Erdem, Jurij Koruza

**Affiliations:** a Institute for Chemistry and Technology of Materials, Graz University of Technology Stremayrgasse 9/Z4 8010 Graz Austria jurij.koruza@tugraz.at; b National Institute for Research and Development of Isotopic and Molecular Technologies 67-103 Donat St. RO-400293 Cluj-Napoca Romania; c Faculty of Engineering and Natural Sciences, Sabanci University, Tuzla Istanbul 34956 Turkey; d Center of Excellence for Functional Surfaces and Interfaces for Nano-Diagnostics (EFSUN), Sabancı University, Tuzla Istanbul 34956 Turkey

## Abstract

The growing need for electroactive components in modern electronics, autonomous vehicles, and miniaturized medical equipment requires new sustainable materials with improved piezoelectric performance. The lead-free perovskite system (Ba,Ca)(Zr,Ti)O_3_ has already been demonstrated to be an excellent alternative to Pb(Zr,Ti)O_3_ for soft-type piezoelectric applications, but its adaptability to hard-type piezoelectric requirements remains unclear. Here, we demonstrate hardening of (Ba,Ca)(Zr,Ti)O_3_*via* targeted defect formation using Fe-acceptor doping. We reveal the underlying defect equations and provide experimental evidence for the formation of 
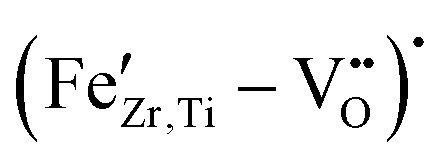
 defect complexes using electron paramagnetic resonance. We describe the influence of Fe-doping on the crystal structure, phase transition behavior, and electromechanical properties, and found outstanding electrostrictive coefficients, up to 0.063 m^2^ C^−4^, in highly doped relaxor-like (Ba,Ca)(Zr,Ti)O_3_. Aging experiments on poled compositions with <1% Fe revealed that piezoelectric hardening through defect complexes occurs in BCZT only after prolonged aging. This resulted in an increase of the figure of merit for resonance applications by over 280% and considerably improved high-power behavior, exceeding the stability of commercial Pb(Zr,Ti)O_3_ materials. Our results demonstrate the large potential of (Ba,Ca)(Zr,Ti)O_3_ for resonant piezoelectric applications and simultaneously give insights into the defect chemistry of acceptor-doped (Ba,Ca)(Zr,Ti)O_3_.

## Abstract 2

Fe–acceptor doping of Ba_0.82_Ca_0.18_Zr_0.08_Ti_0.92_O_3_ piezoceramics induces  defect complexes, which dramatically improve the hard-type piezoelectric properties only after extensive room temperature aging.

## Graphical abstract

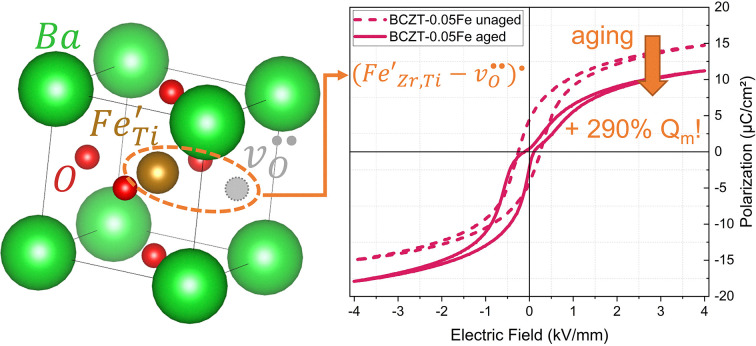

## 1. Introduction

Piezoelectric perovskite ceramics are a crucial class of functional materials with a diverse range of applications, including medical devices, micromechanical systems, and consumer electronics.^1,2^ Due to the lack of lead-free replacements with similar or improved performance, most state-of-the-art technologies continue to use hazardous lead-based Pb(Zr,Ti)O_3_ materials.^3–7^ However, EU legislation, health risks, and environmental concerns have driven fundamental research towards the development of environmentally friendly alternatives, and the first lead-free commercial products are entering the market.^8,9^

(Ba,Ca)(Zr,Ti)O_3_ (BCZT, also abbreviated as (1−*x*)BZT–*x*BCT) is a system derived from the lead-free piezoelectric BaTiO_3_ by substituting 0.3*x* Ca for Ba on the perovskite *A*-site and 0.2(1−*x*) Zr for Ti on the perovskite *B*-site.^10^ It contains compositions that exhibit excellent soft ferroelectric properties, making them suitable for low-frequency applications such as sensors, haptic devices, precision actuators, and medical diagnostic ultrasonic applications.^3,11,12^ However, for high-power transducer applications, which require hard-type piezoelectric materials, BCZT has not been investigated as extensively as other lead-free piezoelectric ceramics, such as NBT- or KNN-based materials.^1^ The classical method to achieve ferroelectric hardening is the introduction of acceptor dopants^3,11–14^ on the perovskite *B*-site. While Fe, Al, Ni, and Mn have been previously reported to harden BaTiO_3_ efficiently,^15–17^ there is limited research on this phenomenon for BCZT. Considering the differences in the electronic and crystal structures, the more complex phase formation sequence, higher sintering temperature, and overall poor homogeneity of BCZT,^18–20^ the fundamental descriptions of doping mechanisms in BaTiO_3_ might not be directly transferable to BCZT.

Initial studies on acceptor doping of the BCZT system were reported by Hansen *et al.*^21,22^ Focusing on dielectric properties and phase transitions, they observed decreases in grain size, *T*_C_, and the *c*/*a* ratio for all investigated acceptors (although Fe was not included in the study). Similar observations were also reported for Fe-doped BCZT by subsequent studies.^23–25^ The behavior has been attributed to the presence of elastic and electric dipoles, resulting from the predicted acceptor–oxygen vacancy defect complexes. Jin *et al.* described the transition of the material to relaxor-like ferroelectric behavior for higher Fe-doping amounts, which has been related to a weakened orbital hybridization of the Ti^4+^ and O^2−^ through Fe^3+^ ions.^23^ Others reported magnetoelectric coupling^24^ and enhanced energy storage properties.^25^

Reports on the possible piezoelectric hardening of BCZT with Fe are rare and, to some extent, contradictory. For example, Jin *et al.* observed no pinching of polarization hysteresis loops and concluded that the interaction between charged defects and polarization must be weak or non-existent.^23^ On the other hand, Gao *et al.*^25^ and Ge *et al.*^26^ reported an increase in the internal bias fields and coercive fields for certain compositions doped with Fe, which they related to hardening by point defects. Hao *et al.* observed a pinching on the polarization hysteresis loops after aging Fe-doped BCZT at 50 °C for two weeks, in their study of the thermal cycling stability of Fe-doped BCZT.^27^ However, no further information on ferroelectric hardening was provided, nor were any resonance parameters reported. These observations underline the need for a systematic investigation of the hardening phenomena in Fe–BCZT and a detailed evaluation of the hard-type piezoelectric properties.

To close this knowledge gap, we first used electron paramagnetic resonance (EPR) and UV-vis spectroscopy to clarify the defect chemical incorporation mechanism for Fe in BCZT, allowing us to experimentally confirm the formation of 
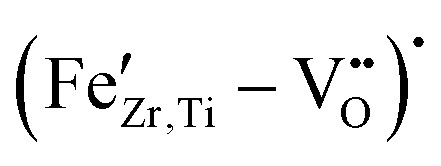
 defect complexes, even in samples that show none of the typical signs for ferroelectric hardening in their electromechanical behavior. Furthermore, we performed room-temperature aging experiments, which demonstrated that the aging of poled Fe-doped BCZT results in asymmetric pinching of the polarization hysteresis, confirming that the classical ferroelectric hardening effect, well-known for BaTiO_3_, also occurs in BCZT, but only after prolonged aging. We then measured the electromechanical performance of Fe-doped BCZT samples under small-signal and high-power resonance conditions and observed a significant improvement in the figure of merit for resonance applications after aging. This finding is a critical advancement towards the future development of hard-type BCZT ferroelectrics for resonance applications.

## 2. Synthesis and methods

Ba_0.82_Ca_0.18_(Zr_0.08_Ti_0.92_)_1−*x*_Fe_*x*_O_3_ (BZT–60BCT–*x*Fe) with Fe concentrations of *x* = 0, 0.0005, 0.001, 0.005, 0.01, 0.02, 0.03, 0.04, and 0.05, were fabricated using the solid-state synthesis method. Note that the tetragonal 60BCT composition has been used instead of the typically investigated 50BCT, to avoid complications in mechanistic descriptions when using multiphase compositions near the polymorphic phase boundaries. High-purity BaCO_3_ (99.95%, Alfa Aesar), CaCO_3_ (99.95%, Thermo Scientific), ZrO_2_ (99.978%, Thermo Scientific), TiO_2_ (99.99%/99.5%, rutile, Thermo Scientific), and Fe_2_O_3_ (99.98%, Carl Roth) powder precursors were used. The particle size distributions of the precursor powders were measured using dynamic light scattering (Cilas 1064) and are reported in Table S1. CaCO_3_, ZrO_2_, and Fe_2_O_3_ were individually pre-milled in ethanol, using a planetary ball mill (Retsch PM400) with 3 mm diameter yttria-stabilized zirconia (YSZ) milling balls for 4 h at 175 rpm. The precursors were dried overnight at 120 °C and weighed in a stoichiometric amount, followed by planetary ball milling of the powder mixtures in ethanol for 4 h at 200 rpm with 3 mm YSZ balls. They were then calcined in Pt-foil-lined Al_2_O_3_ crucibles for 4 h at 1050 °C with a heating rate of 5 °C min^−1^, while the undoped BCZT powder mixture was calcined at 1240 °C. After calcination, the powders were ball-milled again in ethanol with 3 mm YSZ balls at 200 rpm for 4 h.

The dried and sieved calcined powders were pressed into 0.8 g disks. The samples were first pressed uniaxially using a 13 mm die at 75 MPa for 5 min, followed by cold isostatic pressing at 300 MPa for 5 min. Samples were sintered at 1500 °C for 3 h in Pt-foil-lined Al_2_O_3_ crucibles with a heating rate of 5 °C min^−1^ and then furnace cooled. An average relative density of over 94% was achieved for all compositions using this process.

X-ray diffraction (XRD) measurements were performed on calcined powders, as well as crushed sintered samples. Before measurement, the crushed samples were annealed for 30 min at 800 °C with a heating rate of 5 °C min^−1^ and a cooling rate of 3 °C min^−1^. Powder X-ray diffractograms were collected at room temperature in the range of 20° ≤ 2*θ* ≤ 60° using the Miniflex 600 diffractometer (Cu *K*_*α*_, Rigaku), a scanning speed of 5° min^−1^, and a step size of 0.1°. Rietveld refinement of the diffraction patterns was performed using the Jana2020 software^28^ and structures from the Crystallography Open Database (COD). The diffraction peak profiles were fitted using a pseudo-Voigt function.

For microstructural characterization, the sintered samples were ground to a surface roughness of 5 µm using SiC paper and polished to a surface finish of ¼ µm using diamond paste of different gradations. Micrographs of thermally etched samples (etched for 20 min at 1300 °C) were recorded using the Olympus DSX1000 optical microscope. Average grain sizes were determined using a line intercept analysis based on the DIN EN ISO 13383-1 and a correction factor of 1.56.^29^ SEM/EDS of polished, unetched samples, sputtered with a 15 nm carbon layer, was performed on the TESCAN MIRA3 scanning electron microscope with integrated energy-dispersive X-ray spectrometer.

For electron paramagnetic resonance (EPR) spectroscopy measurements, the sintered samples were crushed in an agate mortar and subsequently annealed for 30 min at 800 °C with a heating rate of 5 °C min^−1^ and a cooling rate of 3 °C min^−1^ before measurement. EPR measurements were carried out on a Bruker E-500 ELEXSYS X-band (9.88 GHz) spectrometer at room temperature under identical conditions, using equal amounts of sample.

UV-vis reflectance spectra of the calcined powders were obtained using a Shimadzu UV-2600i spectrophotometer with an ISR-2600 integrating sphere accessory.

The temperature- and frequency-dependent dielectric properties were measured using an impedance analyzer (Alpha-A, Novocontrol) and a heating rate of 2 °C min^−1^. Sintered disk samples were ground to a surface finish of 15 µm on both sides, followed by annealing for 30 min at 800 °C with a heating rate of 5 °C min^−1^ and a cooling rate of 3 °C min^−1^. Silver electrodes, 150 µm thick, were sputtered on both sides, completely covering the circular faces of the samples.

The polarization and strain hystereses were recorded using a modified Sawyer-Tower setup, in which the sample was excited by a high-voltage amplifier (Trek 20/20C) and the strain was measured using an optical displacement sensor (PHILTEC D63-H1QT3). The samples were poled at a field of 4 kV mm^−1^ for 15 min. The piezoelectric coefficient *d*_33_ was measured using a *d*_33_-meter (PiezoMeter System PM100) after aging at room temperature for 24 h.

To investigate the effects of long-term room-temperature aging on the electromechanical properties and formation of defect complexes, the polarization and strain hysteresis of poled samples were re-measured after approximately 8 months of room temperature aging.

Piezoelectric planar resonance measurements were performed on disc-shaped poled samples (after 24 h and 8 months of room temperature aging) using an impedance analyzer and an AC signal of 0.01 V (4192A LF, Hewlett Packard).

The high-power performance of the aged samples was evaluated using a custom-made pulse-drive measurement system employing burst excitation in the planar resonance mode.^30^ Three consecutive measurements were conducted at both resonance and antiresonance for each sample, and the material parameters were derived from the transient decay response.

## 3. Results

### 3.1. Microstructure and crystal structure

**Fig. 1:**
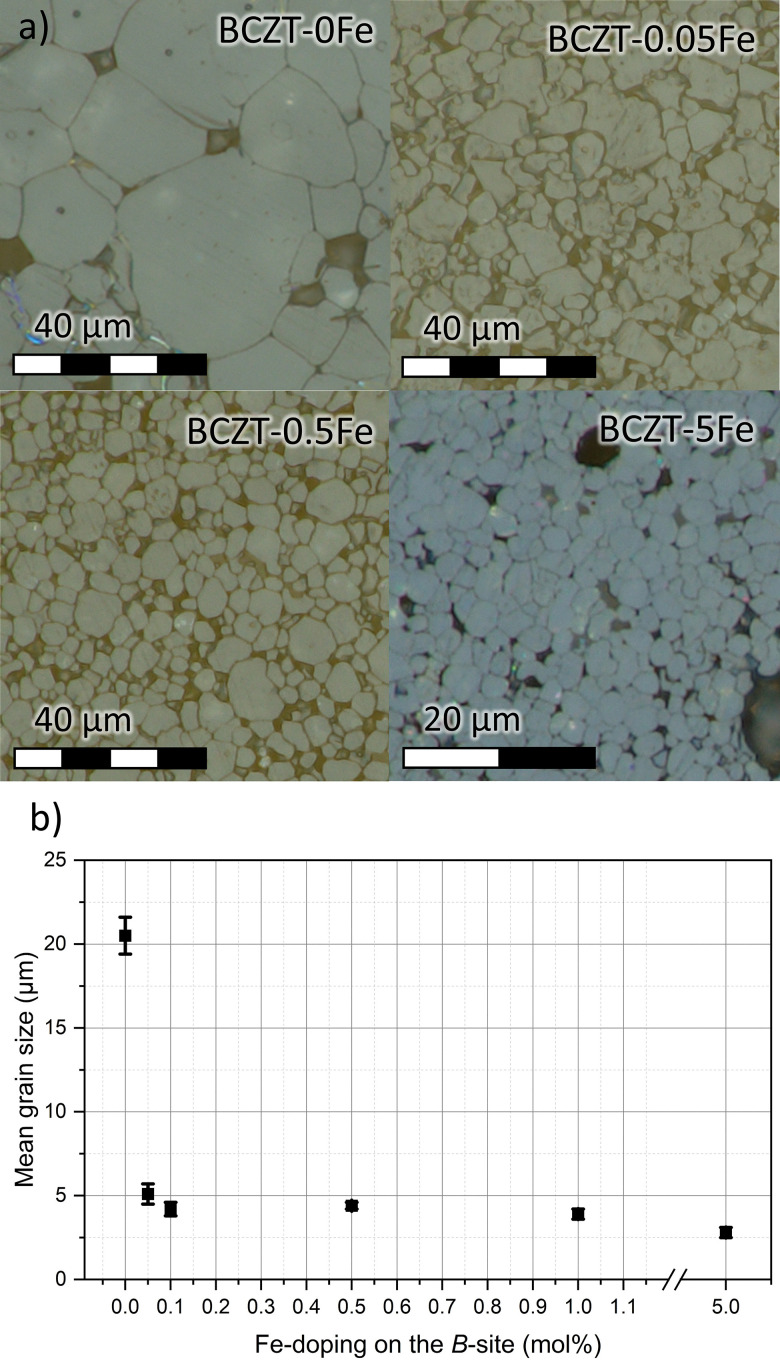
(a) Microstructures of sintered samples, and (b) mean grain size values varying Fe-doping on the perovskite *B*-site.

The micrographs of sintered, polished, and etched samples are presented in Fig. 1a. The average grain size of 20.5 µm, obtained for the undoped BCZT sample, has been strongly reduced to 5.1 µm upon doping with 0.05% Fe nominal dopant concentration (Fig. 1b). A smaller decrease was observed when the Fe content was further increased, with the sample containing 5% Fe having an average grain size of 2.8 µm. None of the samples exhibited abnormal grain growth. It should be noted that the relative density also slightly decreased from 96.8% for pure BCZT to about 95% for the sample with 0.05% Fe, but did not further change with increasing the Fe-content. We note that a reduction of the sintering onset temperature by approximately 150 °C has been observed upon Fe addition, a phenomenon also reported for transition metal doping of BCZT with Mn in the past.^31^

**Fig. 2:**
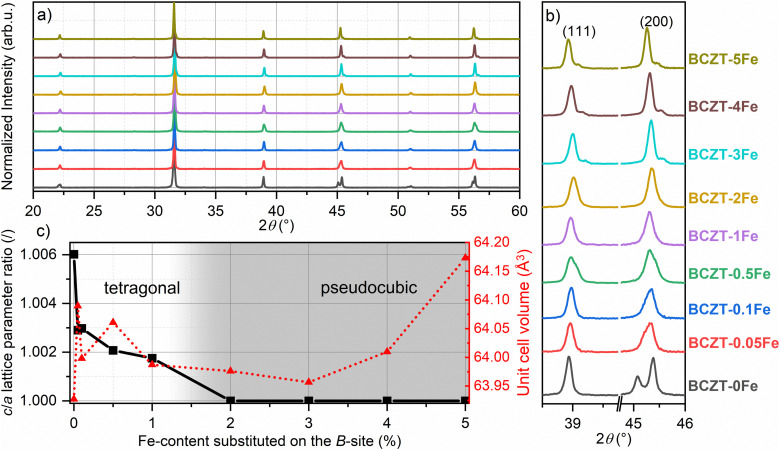
(a) Full-range XRD of sintered, crushed, and annealed samples, (b) enlarged view of 111 and 200 peaks, (c) graph of *c*/*a* and cell volume *vs.* %Fe, whereby the white area denotes the tetragonal ferroelectric region and the gray area the pseudocubic relaxor-like region. The instrumental shift was corrected for the diffractograms shown in (a) and (c), based on the performed Rietveld refinements.

The phase compositions of the sintered samples have been investigated using XRD (Fig. 2). All the samples predominantly consist of the perovskite BCZT phase; however, the secondary phase CaTiO_3_ could be detected in the calcined powders (Fig. S1), and minor amounts are likely also present in the sintered samples (Fig. S2). As demonstrated before, this phase is thermodynamically stable and can thus only be removed by quenching.^18,32^ Furthermore, the XRD analysis revealed the presence of a BaAl_2_O_4_ spinel secondary phase (see Fig. S1–S3), which was introduced due to impurities in the precursor powders. However, we found that this phase does not affect the overall properties of the Fe-doped BCZT material, as shown in the supplementary data. Since the EPR and UV-vis measurements were confirmed to be unaffected by the secondary phase, the defect chemistry of the matrix is most likely not influenced by secondary phases and any observed changes can be attributed to the effects of Fe-doping (see Fig. S4 and S5). We note that the hexagonal phase, which is typically observed in Fe-doped BaTiO_3_,^33^ could not be detected in any of the Fe-doped BCZT samples, neither in the calcined nor in the sintered state.

The single {111}_PC_ reflection and splitting of the {200}_PC_ reflection confirm the tetragonal *P*4*mm* (SG. 99) structure of pure BCZT for the investigated base composition (Fig. 2b), as reported before.^11^ Upon increasing the Fe content, the tetragonality is reduced, and the samples doped with 2% Fe or more exhibit a cubic *Pm*3̄*m* (SG. 221) average structure, while the overall unit cell volume does not change significantly (Fig. 2c). Detailed results of the Rietveld refinement are presented in the SI (Table S2).

### 3.2. Fe-incorporation and point defects

**Fig. 3:**
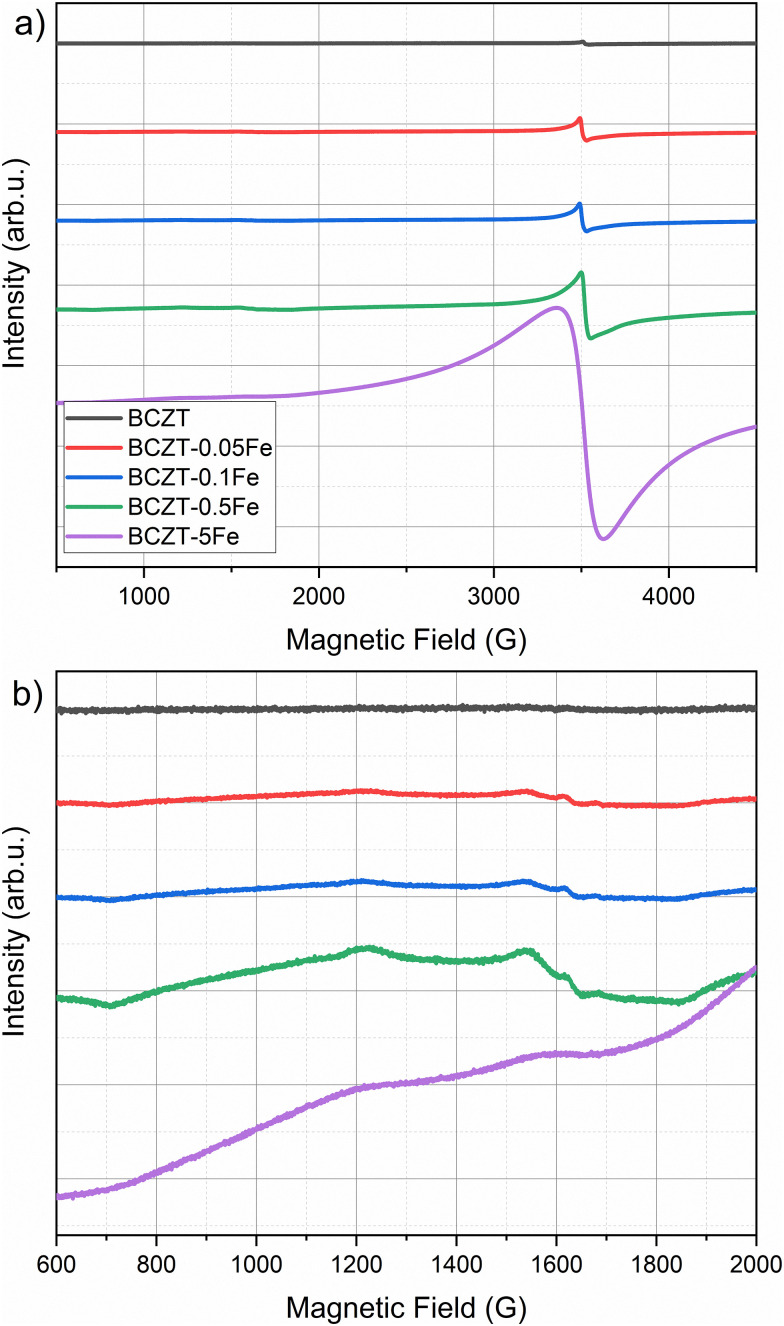
(a) EPR spectra of Fe-doped and undoped BCZT, (b) enlarged details of the broad resonance feature at lower magnetic fields.

EPR spectroscopy is extremely sensitive to the local environment and oxidation state of paramagnetic metal ions in ceramic materials, making it an excellent tool for identifying dilute defect centers that are invisible to most other characterization techniques. For this purpose, the incorporation of Fe into the perovskite lattice has been investigated using EPR spectroscopy. Room-temperature X-band EPR spectra of Fe-doped BCZT, presented in Fig. 3, clearly indicate that Fe is incorporated into the perovskite lattice mainly as high-spin Fe^3+^ on the *B*-site, charge-compensated by oxygen vacancies. The undoped BCZT reference is essentially EPR silent, while already 0.1–0.5 mol% Fe gives a well-defined resonance near 3400 G (*g* ≈ 2.0) together with a broader feature at a lower field (*g* ≈ 4.3), the characteristic fingerprint of Fe^3+^ in a distorted octahedral environment. At the highest Fe content of 5 mol%, the spectra broaden significantly and the EPR intensity no longer scales with the nominal Fe concentration, indicating that only a fraction of Fe remains as isolated 
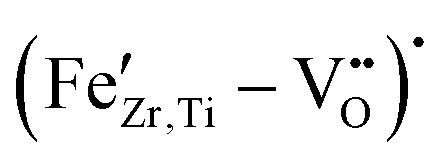
 defect centers while the excess Fe segregates into clustered or EPR-silent environments. A brief explanation of the physical meaning of the *g*-factor and its relevance for identifying oxidation state and local coordination in EPR spectroscopy is provided in the SI.

The detection limit of SEM-EDS prevented an accurate determination of Fe-distribution in samples with low doping concentrations. Exact quantification is prone to error, even for samples with higher doping concentrations. Furthermore, the similar energies of Ba-*L*_*α*_ and Ti-*K*_*α*_ spectral lines reduce the accuracy of the measured *A/B*-site ratio. Nevertheless, we observed that the amount of Fe increases evenly in all phases up to about 3% Fe doping, while for higher concentrations, the secondary phases seem to uptake slightly more Fe than the matrix (Table 1). The measured concentration of Fe in the matrix of the BCZT–5Fe sample was 0.8 mol% Fe, corresponding to *x* = 0.04, which is lower than the nominally targeted *x* = 0.05.

**Table 1:** Amounts of Fe in sintered BCZT samples, as estimated from the SEM-EDS analysis

	Expected Fe in matrix (mol%)	Measured Fe in matrix (mol%)	Measured Fe in BaAl_2_O_4_ (mol%)	Measured Fe in CaTiO_3_ (mol%)
BCZT–1Fe (*x* = 0.01)	0.2	0.2	0.2	0.2
BCZT–3Fe (*x* = 0.03)	0.6	0.5	0.6	0.6
BCZT–4Fe (*x* = 0.04)	0.8	0.6	2.4	0.9
BCZT–5Fe (*x* = 0.05)	1.0	0.8	2.7	1.8

**Fig. 4:**
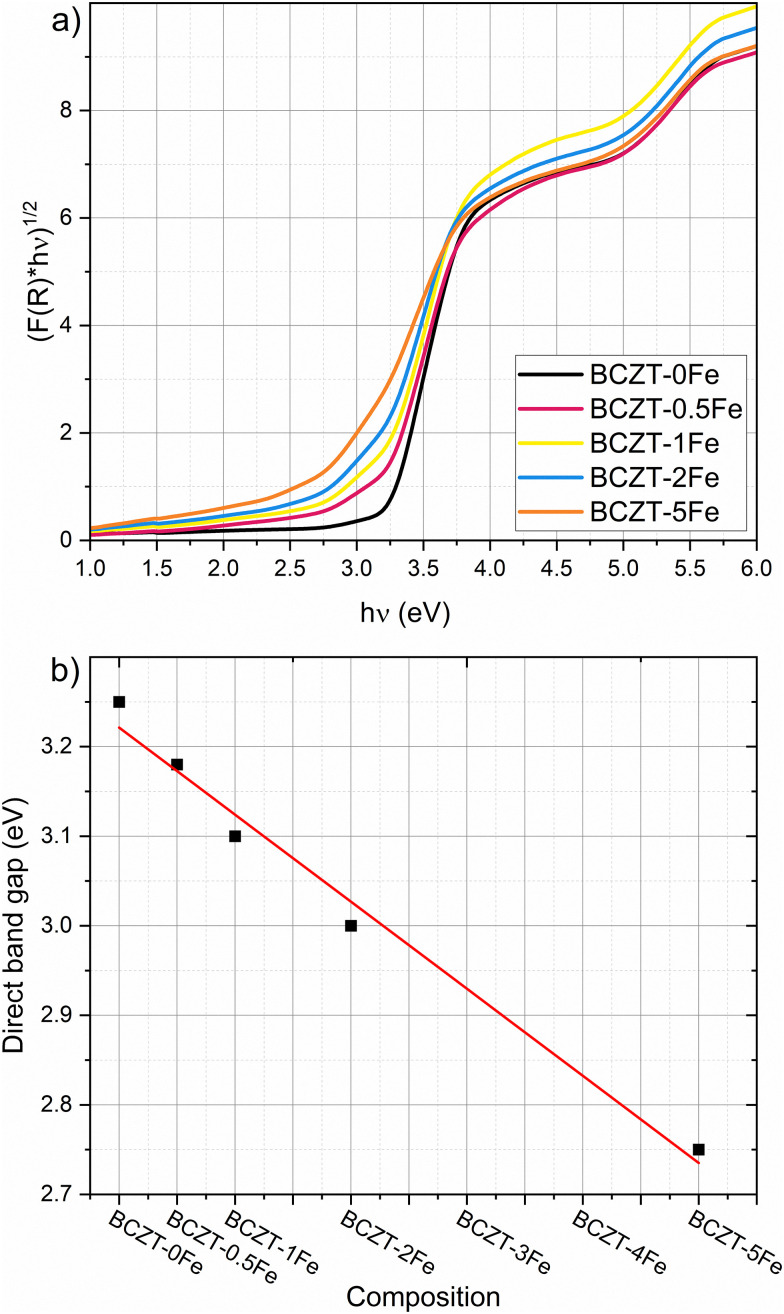
(a) Tauc plot, constructed using reflection data and the Kubelka–Munk function for direct band gaps of Fe-doped BCZT samples. (b) Direct band gaps for different Fe-doping concentrations, estimated graphically from (a), using the tangent method. The additional absorption feature at higher energies may be attributed to the CaTiO_3_ secondary phase and does not impact the band-gap determination.

We determined the optical band gap of undoped and Fe-doped BCZT using UV-vis spectroscopy measurements, employing the Kubelka–Munk function and the tangent method to evaluate the band gap of polycrystalline materials with defects^34^ (Fig. S6). We found that the optical band gap is direct for all investigated compositions (Fig. 4a).^35^ The direct band gap was estimated to be 3.25 eV for undoped BCZT and monotonously decreases with increasing Fe content to 2.75 eV for BCZT–5Fe. Fig. 4b shows the direct band gap as a function of the Fe-doping concentration. Furthermore, a more pronounced Urbach tail emerges with increasing Fe-doping concentration.

### 3.3. Electrical measurements

**Fig. 5:**
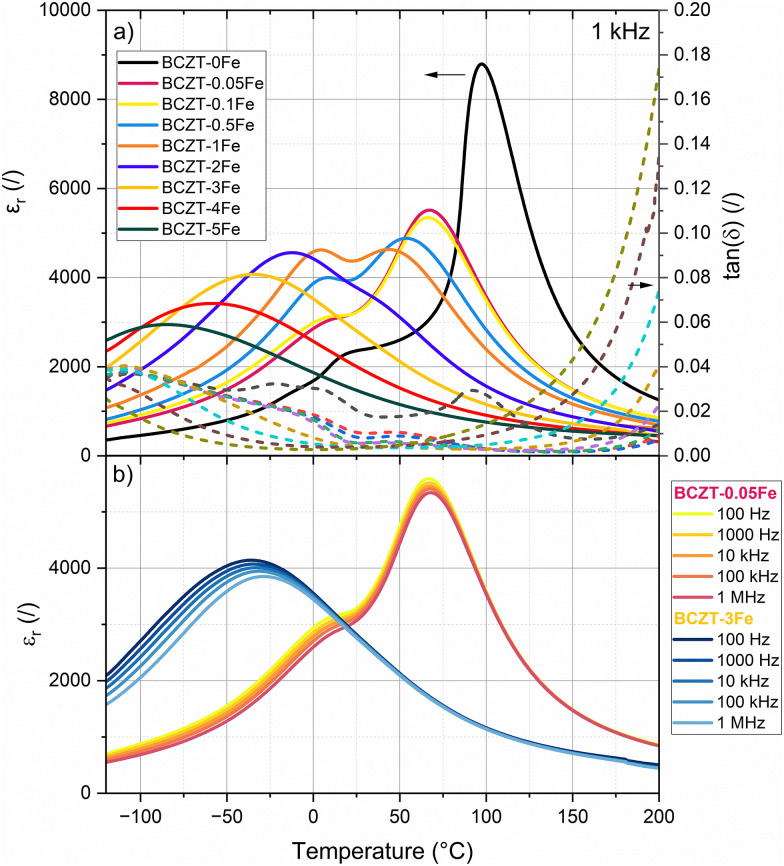
(a) Temperature-dependent permittivity and dielectric loss at 1 kHz, (b) permittivity at different frequencies for the ferroelectric composition BCZT–0.05Fe and for the relaxor composition BCZT–3Fe.

We measured the structural phase transitions of doped and undoped BCZT indirectly through temperature-dependent dielectric permittivity measurements (Fig. 5a) and tracked the temperature shift upon Fe-doping. The anomalies observed in the curve of the BCZT–0Fe sample at −18 °C, 17 °C, and 97 °C have been previously assigned to the rhombohedral–orthorhombic (*T*_R–O_), orthorhombic–tetragonal (*T*_O–T_), and tetragonal–cubic (Curie point, *T*_C_) transitions, respectively.^11^ Our results confirm the tetragonal structure of the BCZT samples with Fe contents up to 1% at room temperature. The *T*_O–T_ and *T*_C_ decrease upon Fe addition, whereby the samples with 2% Fe or more only exhibit one single dielectric anomaly (*T*_m_). A closer look at the permittivity curves measured at different frequencies (Fig. 5b; Fig. S7) revealed a notable frequency dispersion of the dielectric anomalies for the samples with higher Fe-doping concentrations. The frequency dispersion increased with increasing Fe-content. Notably, the room temperature dielectric losses of 0.0204 for BCZT–0Fe are monotonously reduced upon Fe-doping to 0.0084 for BCZT–0.1Fe and 0.0032 for BCZT–5Fe. However, increasing the Fe amount above 0.5% systematically increased the high-temperature dielectric losses.

**Fig. 6:**
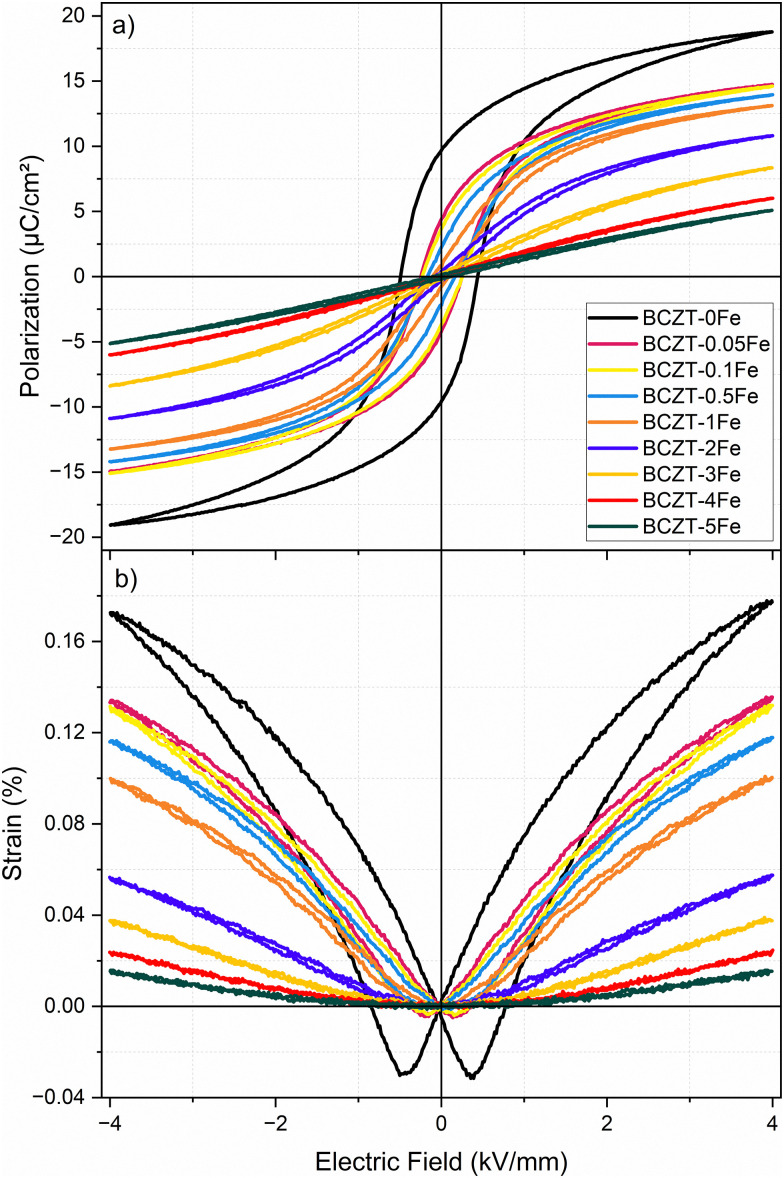
(a) Polarization and (b) strain hysteresis loops of samples (after sintering, no aging), measured at 1 Hz.

The field-dependent polarization and strain hysteresis loops of the Fe-doped samples after sintering (without aging) are presented in Fig. 6, while the characteristic parameters are summarized in Table 2. The dielectric permittivity and piezoelectric charge coefficient *d*_33_ are listed as well. Fe-doping reduced the remanent polarization, coercive field, and strain. The samples with Fe amounts ≤0.5% exhibit typical ferroelectric loops, although the negative strain is very low. On the other hand, the polarization loops of the samples with Fe contents ≥2% are narrow with negligible hysteresis and remanent polarization. The corresponding strain loops exhibit a parabolic shape, typical of electrostriction, as confirmed by the linear increase in strain with the square of the polarization (Fig. S8). The determined *Q*_33_ electrostrictive coefficients are 0.054 m^2^ C^−4^, 0.063 m^2^ C^−4^, and 0.058 m^2^ C^−4^ for the compositions BCZT–3Fe, BCZT–4Fe, and BCZT–5Fe, respectively. These values are comparable to previous reports for Fe^3+^-doped BCT–0.5BZT and are notably higher than those for many other ferroelectric/relaxor ceramic systems.^23,36^

**Table 2:** Overview of characteristic electromechanical parameters for all sintered samples at 25 °C: relative dielectric permittivity (*ε*_r_), dielectric loss tangent (tan(*δ*)), piezoelectric charge coefficient (*d*_33_), coercive field (*E*_c_), remanent polarization (*P*_r_), maximum strain (*s*_max_), and negative strain (*s*_neg_)

	*ε*_r_@1 kHz	tan(*δ*)@1kHz	*d*_33_(pC N^−1^)	*E*_C_ (kV mm^−1^)	*P*_r_ (µC cm^−2^)	*s*_max_ (%)	*s*_neg_ (%)
0% Fe	2358	0.0204	212 ± 11	0.47	9.78	0.186	−0.036
0.05% Fe	3233	0.0105	165 ± 8	0.25	4.37	0.137	−0.005
0.1% Fe	3223	0.0084	163 ± 6	0.25	3.76	0.133	−0.004
0.5% Fe	3993	0.0058	123 ± 2	0.16	2.11	0.119	−0.002
1% Fe	4384	0.0057	11 ± 6	0.10	0.83	0.101	−0.001
2% Fe	3778	0.0044	0 ± 1	0.07	0.35	0.059	−0.001
3% Fe	2798	0.0039	0 ± 0	0.06	0.23	0.040	−0.001
4% Fe	2007	0.0039	0 ± 0	0.07	0.11	0.025	−0.001
5% Fe	1473	0.0032	0 ± 0	0.11	0.17	0.016	−0.002

### 3.4. Aging

**Fig. 7:**
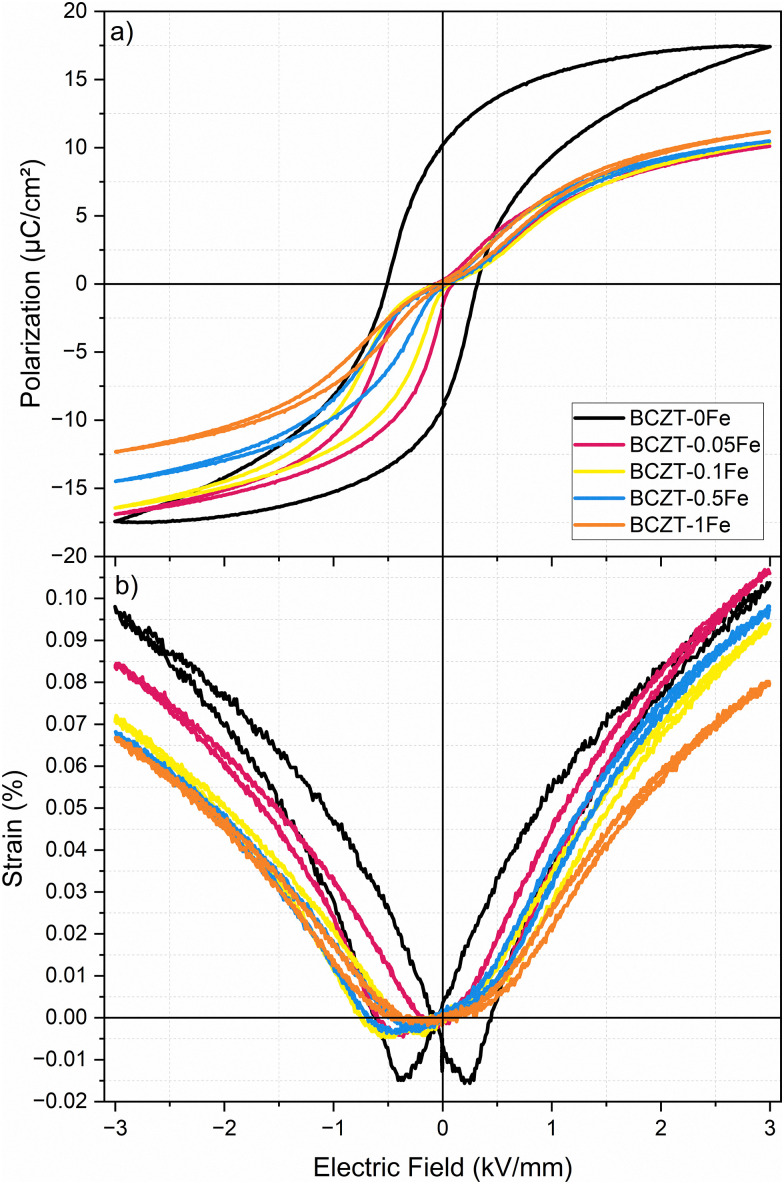
(a) Polarization and (b) strain hysteresis loops of samples (measured after DC poling at 4 kV mm^−1^ for 15 min and 8 months of RT aging), measured at 1 Hz.

Fig. 7 shows the polarization and strain loops of samples doped with up to 1% Fe, which were poled and subsequently aged for about 8 months. The 0Fe sample exhibits a conventional polarization loop, which is slightly shifted along the *E*-field axis. The measured shift corresponds to an internal bias field of *E*_ib_ = 0.076 kV mm^−1^, absent in the unaged samples. On the other hand, the Fe-doped samples show strongly pinched and asymmetric polarization loops, with *E*_ib_ values of 0.437, 0.533, 0.535, and 0.577 kV mm^−1^ for 0.05, 0.1, 0.5 and 1% Fe, respectively (direct comparison between aged and unaged polarization hysteresis loops in Fig. S9), indicating a considerable influence of aging on the sample's properties, compared to those measured within the first few days after sintering. The aged samples also exhibited markedly increased mechanical quality factors (*Q*_m_) and figures of merit for resonance applications (*Q*_m_ × *k*_p_^2^), indicative of piezoelectric hardening (Table 3).

**Table 3:** Overview of characteristic resonance parameters for non-aged and aged ferroelectric samples at RT (*Q*_m_, *k*_p_, FOM)

	Non-aged state			Aged state (8 months)		
	*Q*_m_ (—)	*k*_p_ (—)	*Q*_m_ × *k*_p_^2^ (—)	*Q*_m_ (—)	*k*_p_ (—)	*Q*_m_ × *k*_p_^2^ (—)
0% Fe	150	0.21	6.5	222	0.21	9.8
0.05% Fe	190	0.23	10.4	743	0.20	30.9
0.1% Fe	230	0.21	10.0	905	0.18	28.4
0.5% Fe	500	0.1	5.3	1036	0.14	20.3

## 4. Discussion

### 4.1. Influence of Fe-doping on the microstructure, crystal structure, and electrical properties

Akin to previous reports,^23,26^ we found that *B*-site Fe-substitution considerably reduces the grain size of BCZT (Fig. 1) and lowers the sintering temperature, without the appearance of abnormal grain growth. This indicates a reduction of grain boundary mobility during the sintering process, which has been previously assigned to the presence of oxygen vacancies.^23^ Here, we offer two alternative explanations. Firstly, the mobility of grain boundaries can be strongly inhibited by segregated dopants, a phenomenon known as the solute drag effect.^37^ This effect has recently been experimentally observed in similar systems, namely Fe-doped SrTiO_3_^38^ and Co-doped BaTiO_3_.^39^ Secondly, the diffusion and thus grain growth are also known to be influenced by the concentration of *A*-site vacancies; the latter may be reduced by increasing the number of extrinsic acceptor dopants, because of the lattice electroneutrality condition.^40^ However, since a reduced number of intrinsic *A*-site vacancies is not necessarily the only possible charge compensation mechanism for Fe–acceptor doping, and the solubility of intrinsic Ba-vacancies in the base material is low to begin with, further research is needed to determine the significance of this effect.

**Fig. 8:**
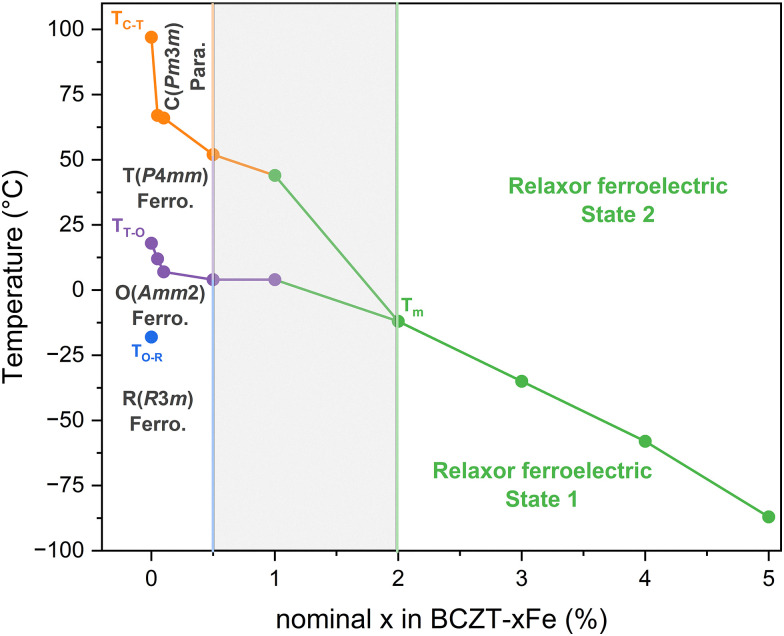
Phase diagram of Fe-doped Ba_0.82_Ca_0.18_Zr_0.08_Ti_0.92_O_3_ constructed from XRD and permittivity data.

The results from our XRD analysis, presented in Section 3.1, show that another major influence of Fe-doping on BCZT is the change of the matrix crystal structure with increasing doping concentration, which results in a reduction of the phase transition temperatures (Fig. 2 and 5) and ultimately in the induction of relaxor-like ferroelectric behavior (Fig. 5b), consistent with previous reports.^21,23,25^ We have created a phase diagram of the different observed ferroic phases, as shown in Fig. 8, based on X-ray diffraction data (Section 3.1) and dielectric measurements (Section 3.3). From the data used to construct the phase diagram, we also calculated the *T*_C_ decrease rate due to Fe-doping. We have identified three regimes for the changes in *T*_C_: a rapid depreciation from 0Fe to 0.05Fe at a rate of −600 °C per %Fe, followed by a more gradual decrease at a rate of −24 °C per %Fe up to 1% Fe, and a higher rate of −33 °C per %Fe for dopant concentrations between 2% and 5% Fe. The initial rapid decrease in transition temperatures from 0 to 0.05% Fe may in part be related to the initial decrease in grain size and will be excluded from further analysis. The main origin of the transition-temperature depression is the reduction of the unit cell distortion, which drops from an initial value of *c*/*a* = 1.006 (for BCZT–0Fe) to *c*/*a* = 1.002 for the sample with 1% Fe. This observation is consistent with previous studies, which reported a decrease in *T*_C_ with lower *c*/*a* in acceptor-doped BCZT.^21^ The decrease rate of *T*_C_ for Fe^3+^-addition in BCZT has been reported to be −37 °C per %Fe by Jin *et al.*,^23^ which is comparable to the rates in our experiment. Hansen *et al.* reported that the decrease rate was related to the oxidation state of the dopant, with the highest rate observed for divalent acceptor ions due to the formation of the greatest number of oxygen vacancies, followed by trivalent and isovalent ions.^21^ Our rates for Fe come close to the rate of −30–50 °C per %Fe, which Hansen *et al.* determined for trivalent ions. The difference could originate from the use of a different BCZT reference composition.

Further insights can be gained from data on Mn-doping in the 4+, 3+, and 2+ oxidation states, reported by Hansen *et al.* Like Fe, Mn is also a transition-metal dopant with a similar ionic radius and charge transition levels within the band gap of BCZT, and might thus show comparable trends to Fe-doping.^21^ Notably, isovalent Mn^4+^-doping did not result in any significant decrease in *T*_C_, despite its smaller size to Ti^4+^, which strongly suggests that the decrease of *T*_C_ in Fe-doped BCZT at a rate of −24/−33 °C per %Fe cannot be explained only by differences in the ionic radii of the respective doping ions, as the ionic mismatch on the *B*-site is smaller for Fe than for Mn.^41^ In summary, the observed reduction in the transition temperatures suggests that Fe is present predominantly in the trivalent oxidation state and that oxygen vacancies are the primary charge-compensating mechanism.

Increasing the Fe-doping amount beyond 1% resulted in a cubic global crystal structure (Fig. 2) and notably changed electrical behavior, displayed by a frequency-dispersion of the permittivity maxima (Fig. 5), reduced polarization and slim polarization hysteresis loops, as well as absence of negative strain and strain loops dominated by electrostriction (Fig. 6). All these features point towards relaxor-like ferroelectric behavior, as previously reported for BCZT with high doping amounts.^23,42,43^ To further verify and characterize the relaxor-like nature, we employed the Vogel–Fulcher relation to determine the freezing temperature of the polar nano-regions.^44,45^ Details of the fitting procedure are provided in the supplementary data (Fig. S10). We found that the BCZT–2Fe, BCZT–3Fe, and BCZT–5Fe follow the Vogel–Fulcher behavior and exhibit a decrease in *T*_VF_ with increasing dopant concentration, from −21 ± 2 °C to −106 ± 4 °C.

High doping levels break the long-range ferroelectric order by disrupting the Ti–O–Ti chains, leading to the formation of polar nano-regions that govern the macroscopic electrical response. This disruption was shown to be more pronounced for heterovalent dopants, since the charge-compensating defects produce strong localized random fields that are more effective in disrupting the ferroelectric order than the purely ionic-size driven disruption in the case of isovalent ions.^46^ The phase transition from ferroelectric to relaxor-ferroelectric behavior also causes a severe deterioration of the macroscopic polarization, evident from the *d*_33_ values listed in Table 2. Since no domain structure emerges in relaxor-type materials above the temperature of the permittivity maximum, *T*_m_, and there is only weak coupling between aligned PNRs, the barriers for polarization switching are significantly lower than in conventional ferroelectrics, which leads to a loss of polarization upon removal of the external electric field.^23,47,48^

### 4.2. Dopant incorporation and defect formation in Fe-doped BCZT

The observed influence of Fe-doping on the microstructure, crystal structure, and electrical properties necessitates a detailed investigation of the defect chemistry and the electronic structure of this system. We determined the direct optical band gap of BCZT to be approximately 3.25 eV, consistent with previous reports.^35^ The oxygen 2p-orbitals predominantly define the valence band maximum of BCZT and lie about 0.1 eV lower than that of BaTiO_3_, due to the addition of Ca-cations.^49,50^ The minimum of the conduction band is formed by the Zr 4d and Ti 3d orbitals, whereby Zr-addition shifts the conduction band edge to higher energies. BCZT typically exhibits p-type conductivity, due to the presence of acceptor-type impurities in most raw materials. Furthermore, nominally stoichiometric BCZT will, during high-temperature processing, exhibit slight BaO-evaporation and consequently the formation of Schottky defects,^51^ resulting in additional cation vacancies that act as acceptors:1
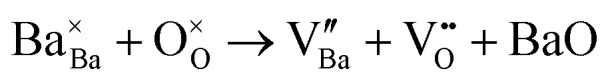


The latter effect can be compensated for by adding excess *A*-site ions during synthesis, which also benefits secondary-phase removal.^18^

Chemical doping of oxides with heterovalent ions triggers various compensating mechanisms, such as ionic or electronic compensation, polaron formation, dopant segregation, or a change in the oxidation state of the dopant itself.^52^ In the case of Fe-doped BCZT, most experimental results point towards ionic compensation *via* the formation of charge-compensating oxygen vacancies:2
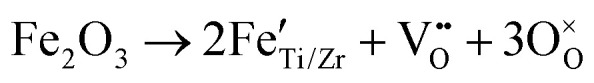


However, it should be noted that other compensation mechanisms, such as Fe segregation observed in SrTiO_3_,^38^ have, in principle, not yet been experimentally excluded.

To clarify the defect formation mechanisms at play, a detailed analysis of the recorded EPR spectra, presented in Section 3.2, is necessary. 0.1–0.5 mol% Fe gives a well-defined resonance near 3400 G (*g* ≈ 2.0), together with a broader feature at a lower field (*g* ≈ 4.3). This pair of signals is the classical fingerprint of Fe^3+^ in a distorted octahedral environment and matches well the 
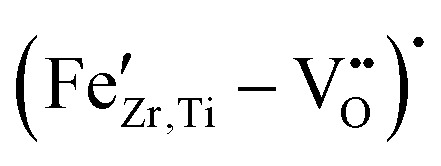
 defect complexes reported in BaTiO_3_, PbTiO_3_, and related perovskites.^53–55^ Within the detection limits of X-band EPR, we do not see clear signatures of Fe^2+^; if present, Fe^2+^ is either EPR-silent in this coordination or below our sensitivity. The absence of detectable Fe^2+^ signals in the X-band EPR spectra is attributed not only to the different electronic configuration of Fe^2+^ compared to Fe^3+^, but also to the presence of large zero-field splitting and fast spin–lattice relaxation in distorted octahedral coordination, which can render Fe^2+^ centers effectively EPR-silent under the present measurement conditions. The positions of the lines do not change with Fe content, indicating that the dominant paramagnetic center remains consistent with an Fe^3+^-type environment across the whole series. With increasing Fe-concentration, the most pronounced evolution in the EPR spectra is the gradual broadening and increase in intensity of the *g* ≈ 2 resonance. Between 0.05 and 0.5 mol% Fe, this signal becomes significantly stronger, reflecting a higher population of isolated 
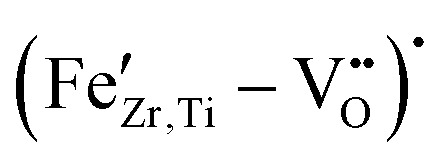
 defect complexes incorporated within the perovskite lattice—an effect consistent with observations in Fe–acceptor-doped BaTiO_3_-based ferroelectrics.^14,55–57^

For high Fe-contents (5 mol%), the EPR spectra broaden and their intensity no longer scales proportionally with the nominal Fe concentration, suggesting that the amount of Fe that is dissolved on the *B*-site and is detectable as isolated 
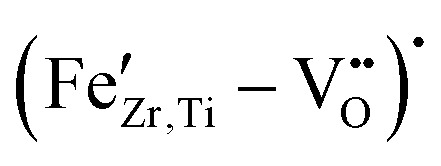
 complexes start to saturate, while the additional Fe goes into other environments. The broad background and derivative-like distortion of the *g* ≈ 2 signal are very similar to what is reported for Fe-rich regions at grain boundaries or Fe-containing secondary phases in other acceptor-modified perovskites.^56^ In other words, EPR results reveal that Fe is occupying the perovskite *B*-site at low doping levels, and most of the EPR-active Fe is lattice-incorporated as 
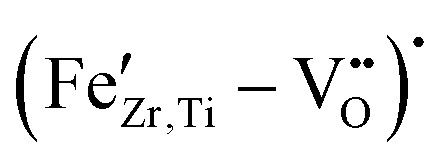
 defect complexes; however, at higher doping levels, a growing fraction of Fe segregates into clustered or non-perovskite-like environments, where strong Fe–Fe interactions make many spins EPR-silent. It should be noted that the EPR features observed at high Fe-contents do not contradict the absence of Fe-related secondary phases in XRD. XRD mainly probes long-range crystalline order and is sensitive only to sufficiently abundant and well-crystallized phases, whereas EPR is a local technique that is highly sensitive to the electronic and magnetic environment of dilute Fe-centers. Consequently, Fe-rich grain-boundary regions, nanoscale clustering, or highly disordered Fe-containing environments may remain invisible to XRD, but still cause strong line broadening and distortion in the EPR spectra. The broad background and derivative-like *g* ≈ 2 features at high Fe contents are therefore attributed to strong Fe–Fe interactions in local Fe-rich environments, rather than to the formation of long-range-ordered secondary phases. This microscopic picture is fully consistent with the literature on Fe-modified BaTiO_3_-based ceramics, where low-level Fe-acceptor doping creates defect dipoles that harden the ferroelectric and improve high-field stability. At the same time, higher dopant levels mainly affect grain-boundary chemistry, conductivity, and domain-wall mobility.^11,14,57^

The similarity between Fe-doped BaTiO_3_ and BCZT is further supported by our UV-vis measurements, detailed in Section 3.2. With increasing Fe concentration, the absorption edge becomes less steep and the Urbach tail increases, indicating more absorption within the band gap, caused by an increased concentration of defects in the matrix, which include 
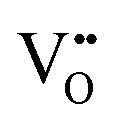
 point defects or 
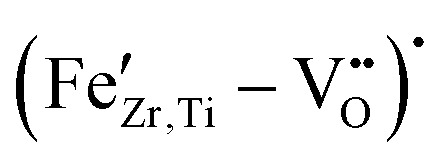
 complexes. Furthermore, the band gap decreases almost linearly with increasing Fe-content (see Fig. 4b), consistent with the literature. This is likely caused by Fe-ions occupying the perovskite *B*-site, which have a lower 3d orbital than the native Ti/Zr-ions.^35^

### 4.3. Ferroelectric hardening can be achieved for Fe-doped BCZT through room temperature aging

In Section 3.3, we presented the *P*–*s*–*E* hystereses of unpoled, non-aged Fe-doped and undoped BCZT samples. Despite an increase of *Q*_m_ upon Fe addition, no sign of ferroelectric hardening was detected in the hysteresis loops, even though we confirmed the presence of 
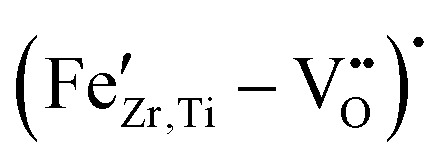
 defect complexes using EPR. We observed a decrease in remanent polarization and coercive field up to 1% and the presence of relaxor properties for higher amounts, consistent with the trends described by others.^23^ While the figure of merit (FOM) for resonance applications increased slightly for small amounts of Fe-doping (Table 3), the respective changes in *k*_p_ and *Q*_m_ could also be explained by the reduced grain size.^58^ This is consistent with the observation of most previous reports, which did not detect hardening in acceptor-doped BCZT. This phenomenon could be explained by considering the study by Hao *et al.*, who investigated the thermal cycling stability of Fe-doped BCZT, aged at 50 °C for 2 weeks. They observed a pinched polarization hysteresis loop, indicating pinning through defect complexes. Notably, Hao *et al.* performed these experiments on the BZT–50BCT composition in which an additional structural phase transition occurs between room temperature and the aging temperature of 50 °C.

To investigate whether a similar behavior can be observed in our samples in the single-phase region, we aged the poled samples for 8 months at room temperature and then re-measured the polarization hysteresis, *d*_33_, and resonance parameters (Section 3.4). After aging, we observed a characteristic pinched and highly asymmetrical polarization loop, similar to that of Fe-doped BaTiO_3_ (Fig. 7). Furthermore, *Q*_m_ increased dramatically, while *k*_p_ only slightly depreciated, leading to an increase in the FOM by 197, 184, and 283% for 0.05, 0.1, and 0.5% Fe-doped BCZT, respectively (Table 3). An increase in the figure of merit was also observed for undoped BCZT, but at a significantly smaller magnitude, compared to the Fe-doped samples. We note that a subsequent experiment using a poled 0.5% Fe-doped BCZT revealed that 10 weeks are sufficient to achieve aging and the samples measured after 8 months can thus be considered aged to saturation. Furthermore, in this subsequent experiment, *Q*_m_ increased by 30% and 50% after 48 h and one week of room temperature aging, respectively, as compared to *Q*_m_ after 24 h aging. While a detailed study on the aging kinetics is ongoing, it is well-known that the reorientation process of defect complexes follows a logarithmic law.^59^ Moreover, samples aged in the unpoled state for up to 5 months did not show any signs of ferroelectric hardening. The slower aging of unpoled samples is also observed in other ferroelectric systems and is attributed to different domain structures.^59,60^

These findings indicate that ferroelectric hardening in Fe-doped BCZT occurs in a manner analogous to that in Fe-doped BaTiO_3_, provided the samples are aged for a sufficient amount of time after poling. However, it is notable that the doped BCZT system required much longer aging times to form asymmetric loops than Fe-doped BaTiO_3_. Note that the presented asymmetric polarization and strain hysteresis curves are typical signatures of piezoelectric hardening by defect dipoles and have been described by the seminal works of Carl and Härdtl in 1978,^59^ as well as Arlt and Neumann in 1988.^16^ The mechanism is based on defect complexes, which form between the acceptor dopant and the charge-compensating oxygen.^61^ A kinetic model for the formation of defect complexes in Fe-doped PbTiO_3_ was proposed by Erhart *et al.*^62^ If Fe^3+^ is incorporated into the BCZT perovskite matrix following eqn (2), statistically distributed oxygen vacancies are initially formed during sintering. After cooling, it is energetically more favourable for the 
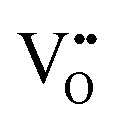
 to position along the *c*-axis of the tetragonal perovskite cell, instead of the *a*-axis, leading to spontaneous re-ordering of unbound oxygen vacancies, followed by the formation of 
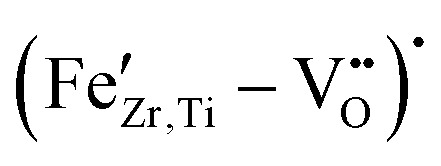
 defect complexes. Hereby the time and temperature needed to reach thermodynamic equilibrium are critically dependent on the energy landscape of the migration barriers for oxygen vacancies between different states. To explain the observed discrepancy between aging in BCZT and BaTiO_3_, this kinetic model suggests that, even though BCZT is derived from the BaTiO_3_ system, the energy landscape of oxygen vacancy migration barriers might be significantly different, increasing the time and temperature required to reach defect equilibrium. The formed defect complexes align with the polarization direction and thus stabilize the ferroelectric domain structure. These complexes can be reoriented by large-enough external electric fields, which is related to the mobility of oxygen vacancies. However, the reorientation of polar defects is strongly time-dependent, due to necessity for oxygen vacancy motion within the crystal lattice (much slower process, as compared to conventional domain reorientation). This introduces an internal bias field within the material, which is macroscopically visible as shifted and asymmetric polarization and strain loops.^63^

**Fig. 9:**
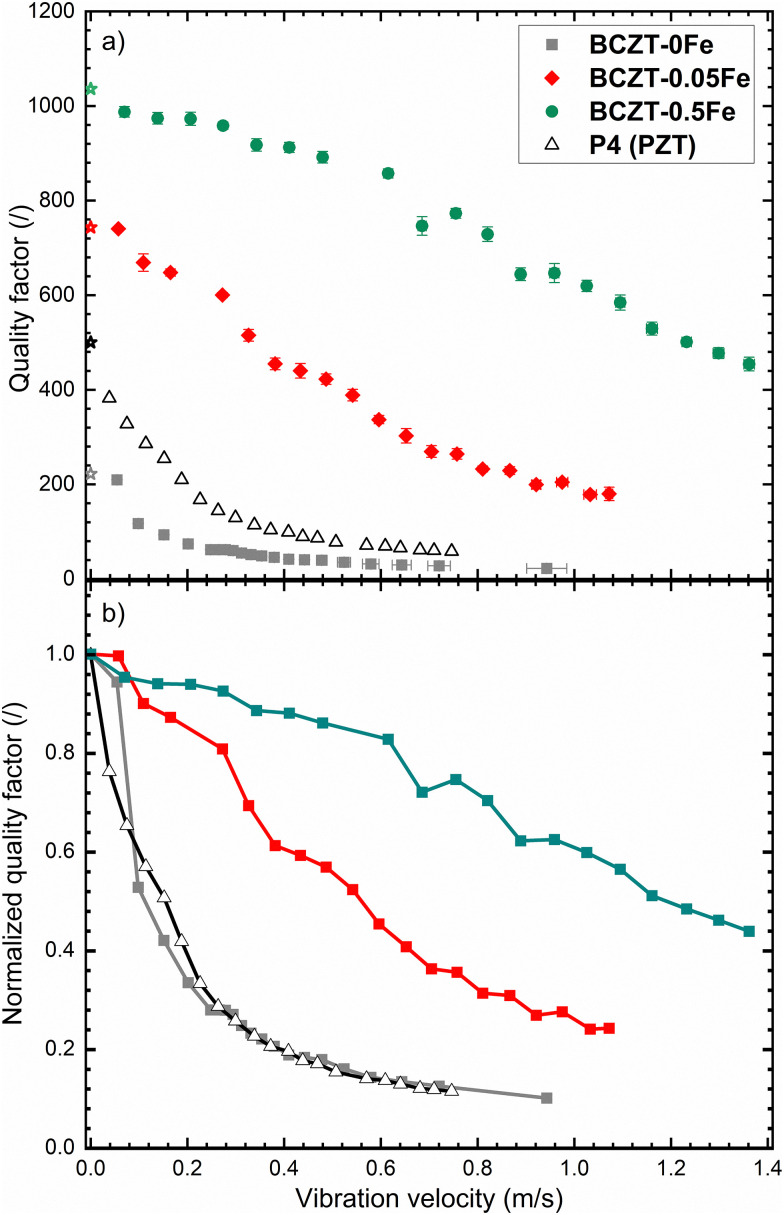
Vibration-velocity dependence of the planar-mode mechanical quality factors of Fe-doped BCZT samples, as compared to commercial medium-hard PZT (P4): (a) absolute values, (b) values normalized to the initial value. The star symbols in (a) mark the small-signal value, while the lines in (b) are added to guide the eye.

Finally, we selected three characteristic samples (0, 0.05, and 0.5% Fe) and evaluated their behavior under high-power resonance conditions (Fig. 9). The results reveal a considerable increase of the *Q*_m_ with Fe-doping over the entire investigated range of vibration velocities. Moreover, the two Fe-doped BCZT samples still exhibit relatively high *Q*_m_ values of 450 and 910 at a vibration velocity of 0.4 m s^−1^, whereas the reference commercial hard PZT sample (Sonox P4, CeramTec, Germany) already dropped to about 100. This prevents the use of PZT at higher vibration velocities, despite the higher electromechanical coupling factors at low vibration velocities, and demonstrates the potential of BCZT materials for extending the operational range of ultrasonic transducers. While the BaAl_2_O_4_ secondary phase, present in similar amounts across all Fe-doped samples, might also contribute to the increased high-power stability overall, the significant improvement observed Fe-doping is increased from 0.05% to 0.5% cannot be explained by the presence of this reinforcement phase.

## Conclusions

In this work, we clarified the defect chemistry and systematically investigated the functional properties of Fe-doped BCZT in both the classical and relaxor-like ferroelectric regimes.

We experimentally confirmed the formation of 
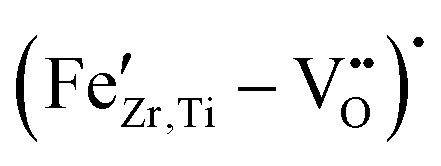
 defect complexes for the first time and discovered that ferroelectric hardening, akin to what is observed in Fe-doped BaTiO_3_, can be achieved after prolonged room temperature aging of poled Fe–BCZT. We determined that this increased the figure of merit for resonance applications by up to 280% and considerably improved the *Q*_m_ stability at high vibration velocities, making the material a promising candidate for high-power resonance applications. Although the absolute values of electromechanical/piezoelectric coefficients reported in this work are still lower than those reported for PZT materials, it should be noted that the base composition used in this study was deliberately picked to be fully tetragonal for ease of analysis and not for optimized functional properties. Choosing a composition closer to the polymorphic phase boundary, such as BZT–50BCT with *d*_33_ values exceeding 500 pC N^−1^, has significant potential to improve performance dramatically. Our results illustrate the critical importance of aging for the accurate assessment of ferroelectric hardening effects. Promising figures of merit for resonance applications were measured, which may be further improved by optimizing the base composition.

Furthermore, we characterized highly Fe-doped BCZT in the relaxor-like ferroelectric regime. The decrease rate of *T*_C_ indicates that Fe is likely still fully incorporated in the 3+ valence state up to 5% Fe-doping, and that the Fe^3+/4+^ charge transition level is not reached. This observation contributes to our fundamental understanding of the electronic structure of Fe-doped BCZT, which is essential for developing systematic doping strategies based on the concept of Fermi level engineering.^52^

## Author contributions

Anna M. Paulik: conceptualization, data curation, formal analysis, investigation, validation, visualization, writing – original draft, Anamaria Mihaljević: conceptualization, data curation, formal analysis, investigation, visualization, writing – review and editing, Kriti Batra: formal analysis, investigation, validation, visualization, writing – review and editing, Arpad M. Rostas: formal analysis, investigation, visualization, writing – review and editing, Emre Erdem: formal analysis, investigation, project administration, writing – review and editing, Jurij Koruza: conceptualization, data curation, project administration, resources, supervision, writing – original draft.

## Conflicts of interest

There are no conflicts of interest to declare.

## Supplementary Material

MA-007-D5MA01411E-s001

## Data Availability

All data relevant to this study is available from the corresponding author upon reasonable request. Supplementary information: precursor powder particle size distributions, analysis results of the phase composition after calcination and sintering (XRD and EDS results), Rietveld refinement results and quality of fit parameters, Kubelka–Munk functions for indirect band gap determination, dielectric permittivity over temperature for each composition at every measured frequency and the corresponding Vogel–Fulcher fits for 2–5% Fe-doping, *S*–*P*^2^ plots for determining the electrostrictive coefficients of 3–5% Fe-doped BCZT, *P*–*E* loops for all aged *vs.* non-aged Fe-doped BCZT samples. See DOI: https://doi.org/10.1039/d5ma01411e.
