# Supplementary material for: Acceptor-doping of lead-free (Ba0.82Ca0.18)(Zr0.08Ti0.92)O3 with Fe induces piezoelectric hardening
Source: Mater Adv. 2026 Feb 19;7(6):3191–203. doi: 10.1039/d5ma01411e (PMC12917726; doi:10.1039/d5ma01411e)
Supplement: MA-007-D5MA01411E-s001 [file MA-007-D5MA01411E-s001.pdf]

Supplementary Information (SI) for Materials Advances.
This journal is © The Royal Society of Chemistry 2026

Supplementary information for

Acceptor-doping of lead-free $(Ba_{0.82}Ca_{0.18})(Zr_{0.08}Ti_{0.92})O_3$ with Fe induces piezoelectric hardening

Anna M. Paulik[1], Anamaria Mihaljević[1], Kriti Batra[1], Arpad M. Rostas[2], Emre Erdem[2,3,4], Jurij Koruza[1,*]

[1]Institute for Chemistry and Technology of Materials, Graz University of Technology, Stremayrgasse 9, 8010 Graz, Austria

[2] National Institute for Research and Development of Isotopic and Molecular Technologies, 67-103 Donat St., RO-400293 Cluj-Napoca, Romania

[3] Faculty of Engineering and Natural Sciences, Sabanci University, Tuzla, Istanbul, 34956, Turkey

[4] Center of Excellence for Functional Surfaces and Interfaces for Nano-Diagnostics (EFSUN), Sabancı University, Tuzla, Istanbul 34956, Turkey

* Corresponding Author.
Adress: Stremayrgasse 9/Z4, 8010 Graz
E-Mail: jurij.koruza@tugraz.at

Table S1. Particle sizes of starting powders (after pre-milling of $CaCO_3$, ZrO_2 and Fe_2O_3).

**Particle size (µm)**	**$BaCO_3$**	**$CaCO_3$**	**ZrO_2**	**TiO_2 (99.5% purity used for undoped BCZT)**	**TiO_2 (99.99% purity used for Fe-doped BCZT)**	**Fe_2O_3**
d_{10}	0.39	0.60	0.34	0.36	0.17	0.18
d_{50}	1.24	1.27	0.89	0.93	0.46	0.49
d_{90}	2.86	2.47	1.61	1.88	0.89	1.01

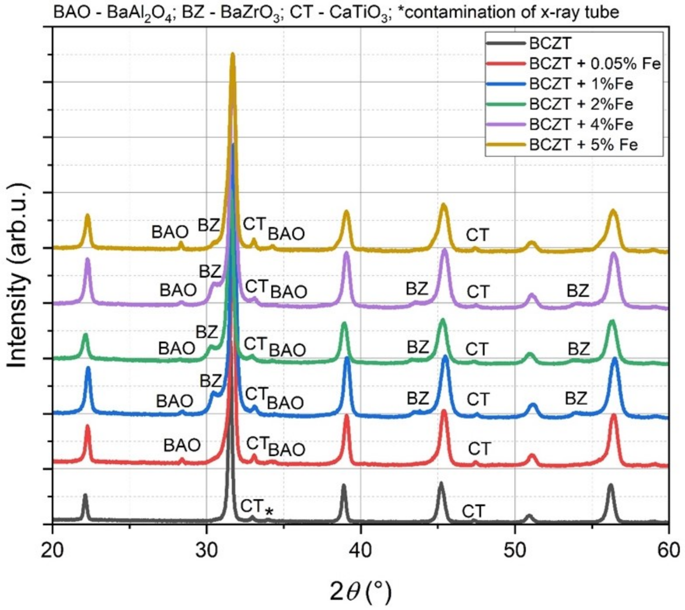

Figure S1. X-ray diffractograms of calcined powders. Pure BCZT was calcined at 1240 °C while all Fe-doped powders were calcined at 1050 °C. The secondary phases $BaAl_2O_4$ and $CaTiO_3$ are marked. Furthermore, some residual $BaZrO_3$ is still present in the Fe-doped samples, which gets fully dissolved during sintering (see **Figure S2** below).

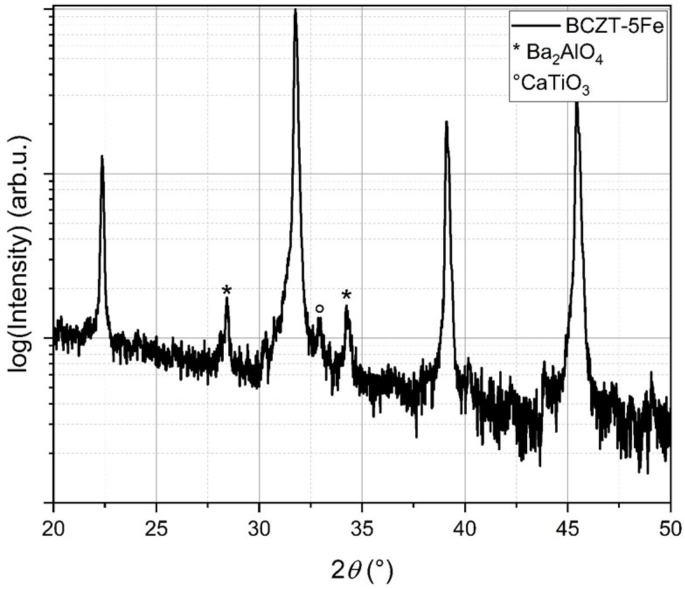

Figure S2. Diffractogram of 5% Fe-doped BCZT after sintering. The $BaAl_2O_4$ and $CaTiO_3$ secondary phases are marked. Note that logarithmic scaling was applied to the *y*-axis for improved visibility.

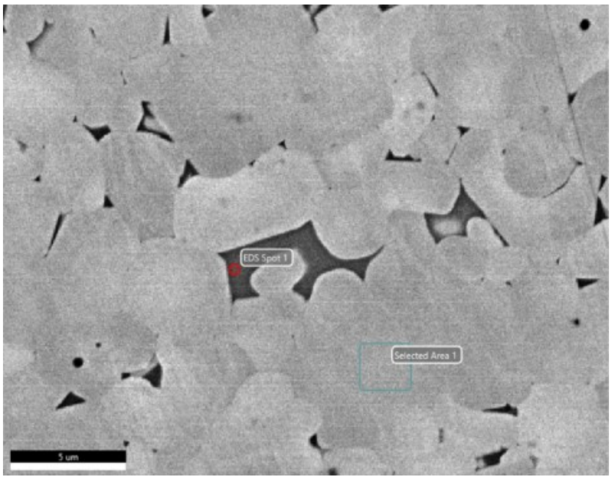

EDS Spot 1 – $BaAl_2O_4$

Element	Weight %	Atomic %	Error %
O	23.9	56.9	7.3
A l	16.3	23.0	6.2
S i	0.5	0.7	12.5
C a	0.9	0.9	16.2
T i	4.2	3.4	8.2
Z r	0.4	0.2	27.8
B a	53.7	14.9	5.3

Figure S3. SEM image of 0.5% Fe-doped BCZT. The corresponding EDS analysis of the secondary phase is shown in the table.

Marak et *al*. studied the influence of Al_2O_3 reinforcement of BCZT and found that $BaAl_2O_4$ inclusions cause a constant offset in dielectric and ferroelectric properties which does not significantly depend on the amount of $BaAl_2O_4$ present or influence the matrix composition [1].

While we found that a $BaAl_2O_4$ secondary phase is present all sintered Fe-doped samples, no significant influence on the optical band gap and EPR response could be determined when compared to a non-contaminated sample, confirming that the matrix defect chemistry is not critically influenced by the presence of $BaAl_2O_4$.

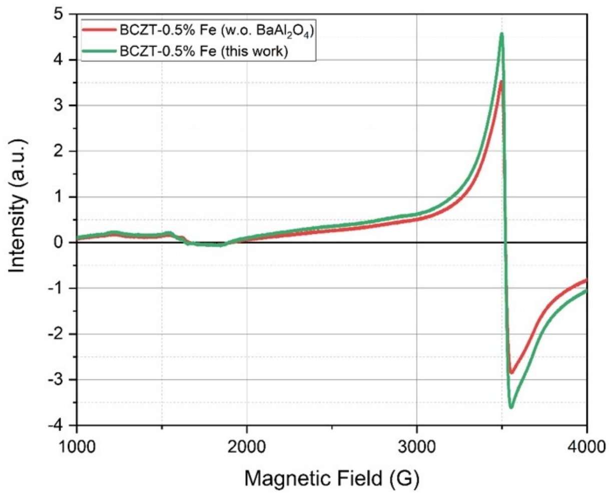

Figure S4. Comparison of EPR spectra for a non-contaminated 0.5% Fe-doped BCZT sample and the 0.5% Fe-doped BCZT sample presented in this work, showing no qualitative differences.

In EPR spectroscopy, the g-factor describes how a paramagnetic electron responds to an external magnetic field. For a free electron, the g-factor is approximately 2.0023. In real solids, however, the g-factor often deviates from this value because the unpaired electron interacts with its local atomic environment through crystal-field effects and spin–orbit coupling. As a result, the g-factor becomes a sensitive probe of the oxidation state, local symmetry, and bonding environment of paramagnetic ions. In transition-metal-doped oxides, such as Fe-doped perovskites, characteristic g-values can be directly linked to specific defect configurations. High-spin Fe^{3+} ($3d^5$, $S = 5/2$) in distorted octahedral coordination typically produces resonances near $g \approx 2.0$ and, in the presence of strong zero-field splitting, additional features around $g \approx 4.3$. These values are widely recognized as fingerprints of Fe^{3+} centers associated with oxygen vacancies in perovskite lattices. Therefore, the observation of specific g-values in EPR spectra provides direct information on the oxidation state and local environment of Fe ions, even when their concentration is too low to be detected by diffraction-based techniques.

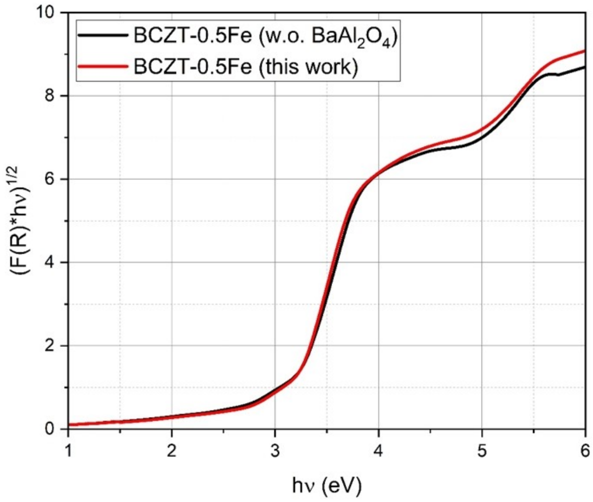

Figure S5. Comparison of the Kubelka-Munk function for a non-contaminated 0.5% Fe-doped BCZT sample and the 0.5% Fe-doped BCZT sample presentejd in this work, showing no qualitative differences.

Table S2. Rietveld refinement results and quality of fit parameters.

Composition	Crystal structure	Lattice constants (Å)		V (Å^3)	*GOF*	R_p	R_{wp}
		a	*c*				
**BCZT-0Fe**	Tetragonal (*P4mm*)	3.9905	4.0145	63.9	1.13	5.04	6.54
**BCZT-0.05Fe**	Tetragonal (*P4mm*)	3.998	4.0096	64.1	1.07	6.05	8.75
**BCZT-0.1Fe**	Tetragonal (*P4mm*)	3.996	4.0079	64	1	5.56	8
**BCZT-0.5Fe**	Tetragonal (*P4mm*)	3.9985	4.0068	64.1	0.92	5.03	7.36
**BCZT-1Fe***	Tetragonal (*P4mm*)	3.9974	4.0044	64	1.19	3.93	6.11
**BCZT-2Fe***	Cubic (*Pm3m*)	3.9995	3.9995	64	1.41	5.03	7.25
**BCZT-3Fe**	Cubic (*Pm3m*)	3.9991	3.9991	64	1.44	5.44	7.69
**BCZT-4Fe**	Cubic (*Pm3m*)	4.0002	4.0002	64	1.36	4.84	6.98
**BCZT-5Fe**	Cubic (*Pm3m*)	4.0036	4.0036	64.2	1.17	6.86	9.1

* BCZT-1Fe and BCZT-2Fe show similar parameters for both structures; the table gives the one with the slightly better fit.

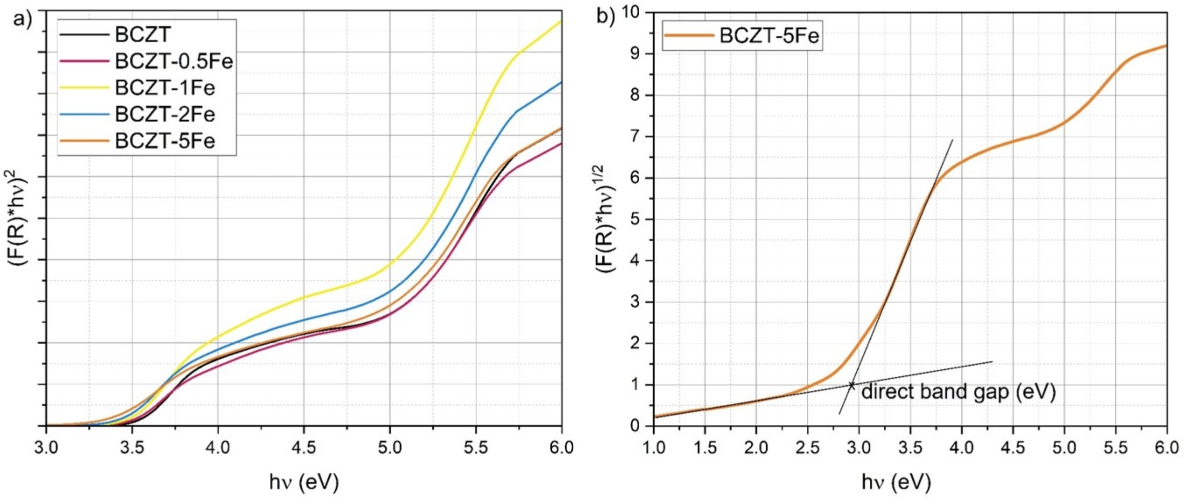

Figure S6. a) Kubelka-Munk function for indirect band-gap determination, b) schematic depiction of the tangent method of determining bandgaps of polycrystalline materials with defects.

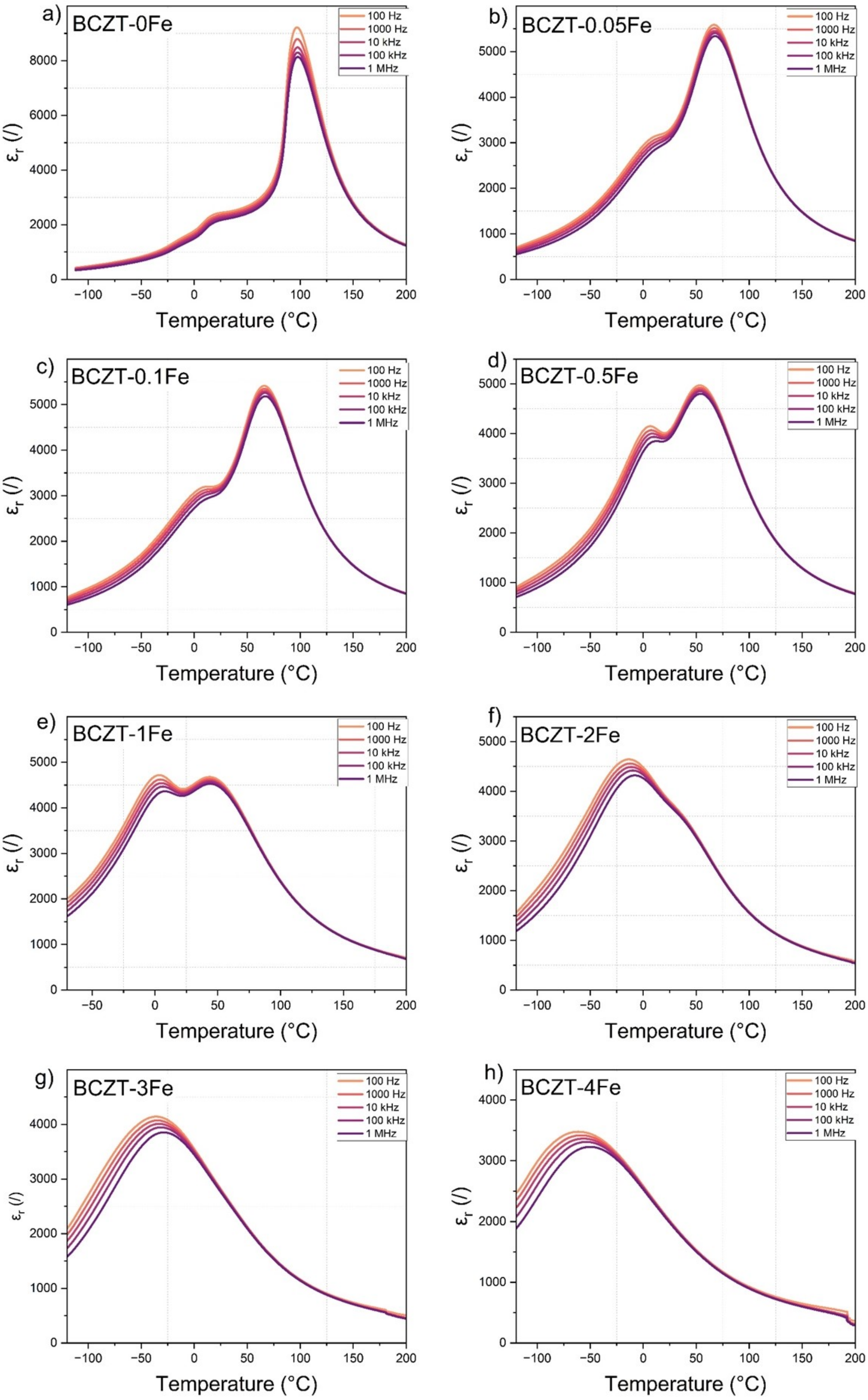

Figure S7. Frequency dependence of T_C observed in the dielectric permittivity, measured for BCZT-0Fe … BCZT-4Fe.

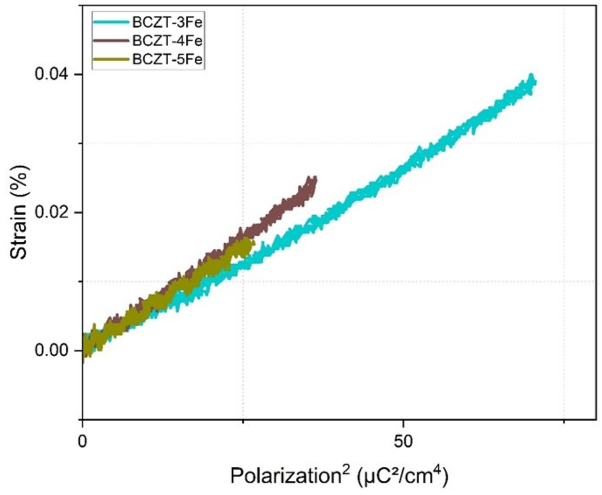

Figure S8. S-P^2 plot of the relaxor-like ferroelectric BCZT-3Fe, BCZT-4Fe and BCZT 5Fe compositions for determination of the electrostrictive coefficients.

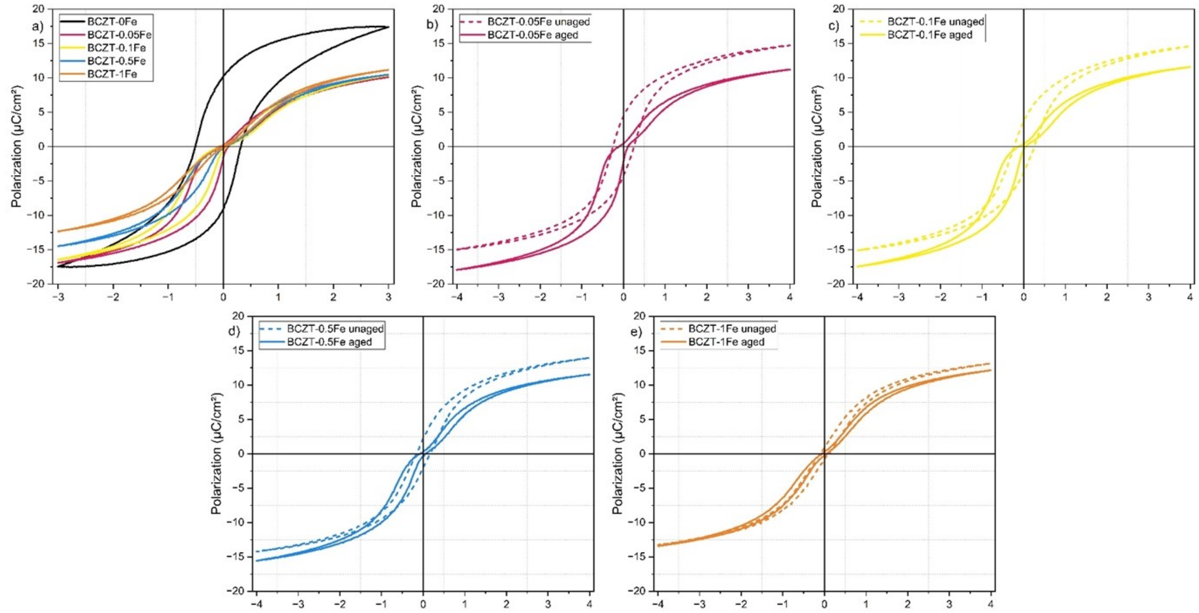

Figure S9. Comparison of polarization loops between aged and non-aged samples of the same composition.

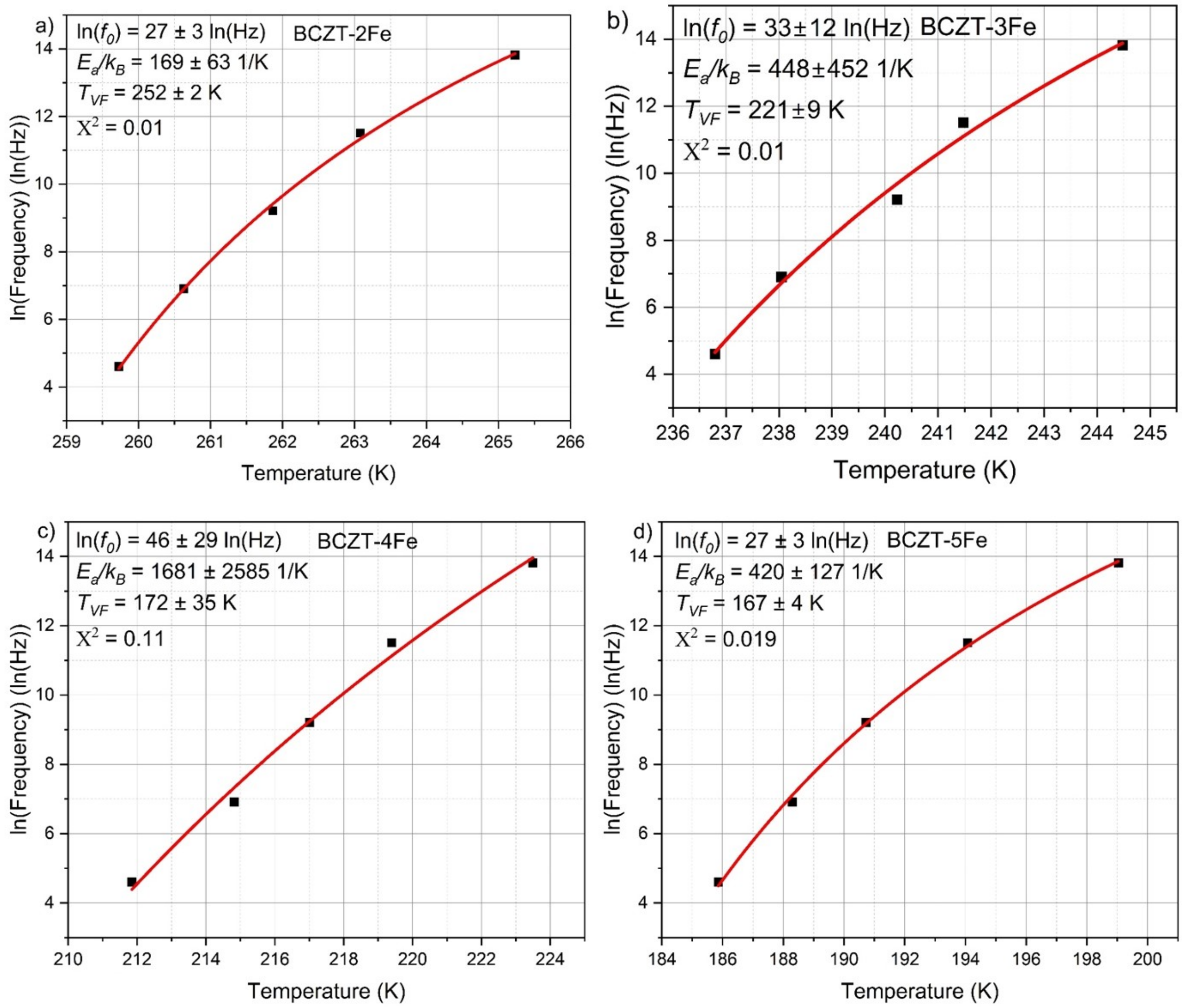

Figure S10. Vogel-Fulcher fits of relaxor-ferroelectric Fe-doped BCZT compositions including fit parameters

References in Supplementary information:

[1] V. Marak, D. Drdlik, V. Bijalwan, P. Tofel, J. Erhart, Z. Chlup, K. Maca, Effect of alumina or zirconia particles on the performance of lead-free BCZT piezoceramics, Ceramics International 50(24, Part A) (2024) 53491-53501.
